# 

**Published:** 1869-07-15

**Authors:** 


### CONTENTS.

TRANSLATIONS.

Anatomical and Physiological Observations made upon Decapitated Subjects. By M. Ch. Robin ..... 430

COMMUNICATIONS.

Super-Oxygenation in the Dynamics of Organic Life ..... 441
Rush Medical College ..... 445
Medical College Fees ..... 447

SOCIETY REPORTS.

Chicago Medical Society ..... 448
Carroll County Medical Society ..... 449
Minnesota State Medical Society ..... 450
Annual Meeting of the Fillmore County, Minn., Medical Society ..... 451

EDITORIAL.

Rush Medical College ..... 442
Medical College Fees ..... 452
A Gratifying Appointment ..... 253

NEWS ITEMS ..... 455

BOOKS RECEIVED ..... 460

